# Navigating rare disease medications: A pharmacist’s primer

**DOI:** 10.1177/17151635241228274

**Published:** 2024-01-30

**Authors:** Ahmad Shakeri, Yasmin Abdul Aziz, Mina Tadrous

**Affiliations:** Leslie Dan Faculty of Pharmacy, University of Toronto; Women’s College Research Institute, Women’s College Hospital, Toronto, Ontario; Leslie Dan Faculty of Pharmacy, University of Toronto; Leslie Dan Faculty of Pharmacy, University of Toronto; Women’s College Research Institute, Women’s College Hospital, Toronto, Ontario

In 2019, more than half, or 56%, of the drugs approved by Health Canada were for patients with rare diseases.^1,2^ These drugs not only present clinical complexities but also pose challenges regarding the evidence used in their development, reimbursement structures and patients’ and pharmacies’ access to them. In March 2023, the Ministry of Health announced measures in support of the first-ever National Strategy for Drugs for Rare Diseases, investing up to $1.5 billion with provinces and territories over 3 years, ultimately aiming to increase access to and affordability of effective drugs for rare diseases and leading to the increased prevalence of these medications across Canadian pharmacies in the near future.^3^ This commentary aims to provide introductory information to help pharmacists understand the current landscape of rare diseases in Canada, to ensure patients receive the optimal support they need from their pharmacy team.

## Defining rare diseases

Health Canada defines rare diseases as life-threatening, debilitating, or serious and chronic conditions that affect a small number of individuals with limited or no available treatments.^4^ More specifically, the Canadian Organization for Rare Disorders defines rare diseases as conditions that affect fewer than 1 in 2000 people.^5^ With ongoing advancements in genomics and precision medicine, the number of documented rare diseases is expected to continue increasing.^6^ Thus, while individual rare diseases may have a low occurrence, when combined, it is estimated that 8% of Canadians live with them in their lifetime.^1^ Given that pharmacists are the most accessible and frequently visited health care professionals, it is essential for the profession to be well informed, knowledgeable about such medications and comfortable in assisting patients living with rare diseases.

## Rare disease medications

### Clinical and safety uncertainty

To obtain rare disease drug approval through Health Canada, substantial evidence of clinical safety and efficacy is necessary. Clinical trial design requires an appropriately powered sample size, a control group and clinically meaningful outcome measures. For rare diseases, this is not always feasible; for example, the power of a clinical trial is maximized by increasing the number of subjects, but this is not always achievable in rare diseases due to practical and ethical limitations, especially in pediatric populations.^7^ Pharmacists need to be mindful of various clinical considerations when caring for patients with rare diseases, as many of these drugs lack robust clinical evidence in the literature. Frequently, these drugs have undergone small-scale clinical trials, resulting in insufficient evidence compared with other medications on the market. Real-world evidence from prospective cohorts and registries plays a significant role in evaluating these drugs. Consequently, many patients participate in registries to address the research limitations inherent in the clinical trials of rare diseases.

The safety profiles of these drugs may also be uncertain due to the limited number of patients using them. Therefore, pharmacists need to adjust their monitoring approach for these patients, modifying follow-up schedules and identifying unique drug therapy problems and side effects. Furthermore, many of these medications require specialized administration methods, often necessitating patients to visit specialty clinics for treatment. It is important for pharmacists to have a thorough understanding of the various drug delivery methods since several of these are administered via intravenous injection or infusion. This highlights the significance of educating patients about the administration process and the potential outcomes, such as infusion-related reactions that are common with monoclonal antibodies. Last, given the nature of these medications, which may have limited efficacy and safety evidence in the literature, pharmacists should maintain close communication with physicians to ensure a comprehensive understanding of the patient’s experience with the medication.

Most rare disease medications cannot be obtained from a local pharmacy. Pharmaceutical companies often have partnerships with a limited number of specialty pharmacies that dispense the drug. Therefore, pharmacists should be aware that some of their patients may use specialty pharmacies while still requiring their care for other medications. Typically, there are 2 pathways for a prescription of a rare disease medication. First, in the specialized medical clinic, patients receive the diagnosis and prescription. The clinic may have designated personnel responsible for handling the prescription and guiding patients to the specialty pharmacy. In this case, the pharmacist should be aware that the patient may be receiving the medication elsewhere based on guidance from the clinic, even though the patient obtains other medications from them (i.e., the pharmacist was not involved in the prescription process). Second, the patient may receive a prescription that gets rejected when the pharmacist attempts to claim it because it must be filled at a specialty pharmacy. In such cases, the pharmacist must navigate the situation appropriately.

### Cost and access

Generally, treatments for many rare diseases are scarce or nonexistent. When available, they are prohibitively expensive, ranging from $100,000 to $2,000,000 per year and often required for the patient’s lifetime.^8^ As of 2019, approximately 73% of drugs for rare diseases cost more than $200,000 per year.^1,9^ Moreover, the complex processes required to bring these drugs to Canadian patients can cause delays in patient access to these drugs, resulting in extended patient suffering due to needs not being addressed in a timely manner. Notably, submissions to Health Canada for 84% of these drugs approved between 2002 and 2016 were filed after those submitted to the Food and Drug Administration and the European Medicines Agency, with a median delay of 253 days.^10^

Once Health Canada authorizes these drugs for sale, both public and private drug plans can decide the conditions of drug coverage for their members. Public (government) drug plans aim to enhance medication access for patients by covering specific populations, such as seniors, children, individuals with low incomes, or those with serious medical conditions. Instead of providing general approval for high-cost drugs used in rare diseases, public drug plans have prior authorization programs, like the Exceptional Access Program in Ontario and Alberta Rare Diseases Drug Program, to assess each patient’s case individually. They impose specific conditions for coverage based on additional clinical information provided by the prescribing physician. Likewise, private drug plans, including employer-sponsored plans, make independent decisions regarding coverage, and these decisions may vary from other private plans and public drug plans.

In cases in which a patient could benefit from a rare disease drug not authorized for sale in Canada, physicians may submit requests to Health Canada’s Special Access Program (SAP) or Patient-Support Programs (PSPs). The SAP enables physicians to access drugs approved in other jurisdictions outside of Canada to treat patients with rare diseases when other therapies have failed. These drugs can be provided free of charge, and if there is a cost involved, it may be covered by public or private drug plans or, in some cases, by the patients themselves. On the other hand, PSPs, sponsored by pharmaceutical companies, supply drugs to select patients on a compassionate basis, without guaranteeing continued access to these medications. This compassionate supply aims to provide access to a drug in cases where it is not covered by public or private drug plans.

## Conclusion

The prevalence of drugs for rare diseases is increasing, with their integration into community and in-patient practice settings across Canada becoming more common. This requires pharmacists to collaborate closely with patients to navigate this unique therapeutic landscape, particularly concerning access and affordability. ■
